# Risk of Injury to the Superficial Branch of the Radial Nerve in Dorsal and Anterolateral Approaches to the First Carpometacarpal Joint

**DOI:** 10.1177/15589447251392940

**Published:** 2025-12-17

**Authors:** Irene Pérez de Gracia-Velázquez, Javier De Torres-Urrea, Olga Roda, Clarisa Simón-Pérez, Natividad Martín-Morales, Francisco O’Valle, Pedro Hernández-Cortés

**Affiliations:** 1 Granada University, Spain; 2 Mediterráneo Hospital, Almeria, Spain; 3 Valladolid University, Spain; 4 University Hospital of Valladolid, Spain; 5 Instituto de Investigación Biosanitaria (IBS), Granada, Spain; 6 University Hospital of Granada, Spain

**Keywords:** trapeziometacarpal osteoarthritis, trapeziometacarpal prosthesis, surgical approach, superficial branch of the radial nerve, nerve ending density, surgical wound

## Abstract

**Background:**

Complications of thumb basal joint arthritis surgery include surgical wound pain and *cheiralgia paresthetica* through involvement of the radial nerve superficial branch (RNSB). The aim of this study is to compare the risk of nerve injury between anterolateral and dorsal approaches to the first carpometacarpal joint (1CMCJ) by measuring the distance between the incisions and RNSB, recording crosses between them, and evaluating the density of skin nerve endings at incision sites.

**Methods:**

In this descriptive study of 20 cryopreserved cadaver specimens, the anatomical distribution of the RNSB and its relationship with 1CMCJ anterolateral and dorsal approaches were determined by macro-dissection, and histomorphological analysis and digital imaging were used to measure cutaneous nerve ending density at the 1CMCJ and incision sites.

**Results:**

In comparison to dorsal approach, the minimum distance from the RNSB was significantly shorter (1.30 ± 1.94 mm vs 3.70 ± 2.71 mm), and the total number (16.60 ± 8.50 vs 9.97 ± 7.51) and density (2.00 ± 0.83 vs 1.29 ± 0.77 mm^2^) of nerve endings were significantly higher with anterolateral approach. With the anterolateral approach, more nerve structures were observed in the distal *versus* proximal section of the incision, although the difference was only close-to-significant.

**Conclusion:**

The incision is closer to the RNSB pathway with Wagner’s anterolateral approach than with Gervis’ dorsal approach, crossing with nerve branches in 50% of cases. The density of nerves is higher in the skin overlying the anterolateral *versus* dorsal aspect of the carpometacarpal joint. The risk of neuropathic wound pain after 1CMCJ surgery could be higher with the anterolateral approach, which should therefore be avoided.

## Introduction

Complications of open or arthroscopic surgery of the first carpometacarpal joint (1CMCJ) include wound pain and complex regional pain due to involvement of the radial nerve superficial branch (RNSB).^1-5^ However, their prevalence among patients undergoing thumb basal joint arthritis surgery is not always reported in published case series. We hypothesized that the site of the surgical wound might influence the risk of these complications.

Open surgery of the 1CMCJ is usually performed via a dorsal or anterolateral approach.^6^ In the dorsal approach, described for trapeziectomy by Gervis in 1949,^7^ the incision extends from the proximal two-thirds of the first metacarpal to the distal third of the trapezium, posing a potential risk to the integrity of the radial artery and/or RNSB.^8^ In the anterolateral approach, proposed for Bennett’s fracture-dislocation by Wagner in 1950,^9^ the incision is performed at the junction between palmar and dorsal skin overlying the 1CMCJ, and it also involves a potential risk of RNSB injury and postoperative dysesthesias.^6^ To our best knowledge, no published study has compared the risk of RNSB injury between dorsal and anteroposterior approaches to the 1CMCJ.

The objective of the present descriptive study in cadaver specimens was to compare the risk of iatrogenic nerve injury and surgical wound pain between dorsal and anterolateral approaches to the 1CMCJ, examining the proximity of incisions to the RNSB pathway and using histomorphometry and digital imaging analysis to compare the density of skin nerve endings at incision sites.

## Materials and Methods

This anatomical and histomorphometric descriptive research study was approved by the Biomedical Research Ethics Committee of Andalusia in April 2023 (Annex I; supplementary material). All studied individuals had given their informed consent to the utilization of their remains for scientific purposes.^10,11^

The study used 20 forearm-and-hand specimens (11 left-side, 9 right-side) from 15 cryopreserved cadavers (7 men) aged between 52 and 78 years at death. No specimen had any history of wrist surgery or injury. All specimens were defrosted at room temperature and dissected in the Department of Human Anatomy of the University of Medicine of Granada (Spain) between October and December 2023.

### Anatomical Study

**Figure 1.**
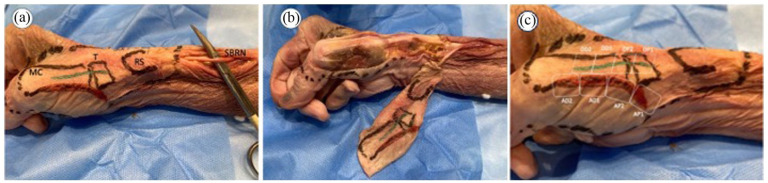
Cadaver specimen dissection. *Note.* (a) Marking on the skin of the MC, T, and RS. Drawing of the anterolateral (red line) and posterior (green line) approaches and identification of the sensory branch of the RNSB above the wrist. (b) Elevation of the skin and subcutaneous cellular tissue overlying the affected anatomical region in continuation with the RNSB. (c) Division into segments for morphometric study of the dorsal and anterolateral approaches. In both incisions, samples were taken from 2 proximal segments (AP1 and 2 for the anterolateral approach and DP1 and 2 for the dorsal approach) and 2 distal segments (AD1 and 2 and DD1 and 2, respectively). MC = metacarpal; T = trapezius; RS = radial styloid; RNSB = radial nerve superficial branch; AP1 = first proximal segment in anterolateral approach; AP2 = second proximal segment in anterolateral approach; DP1 = first proximal segment in dorsal approach; DP2 = second proximal segment in dorsal approach; AD1 = first distal segment in anterolateral approach; AD2 = second distal segment in anterolateral approach; DD1 = first distal segment in dorsal approach; DD2 = second distal segment in dorsal approach.

On 10 specimens, indelible ink was used to mark the styloid radial process, trapezium, first metacarpal bone, and the theoretical incisions for anterolateral and dorsal approaches to the 1CMCJ. An incision was performed in the lateral aspect of the forearm to identify the RNSB (Figure 1a).

A scalpel was used to elevate the skin of the radial aspect of wrist and hand en bloc with the subcutaneous cell tissue (Figure 1c). The RNSB was dissected with the subcutaneous cell tissue facing upward under ×3.5 magnification loupes, individualizing and labeling each branch with black Indian ink.

**Figure 2.**
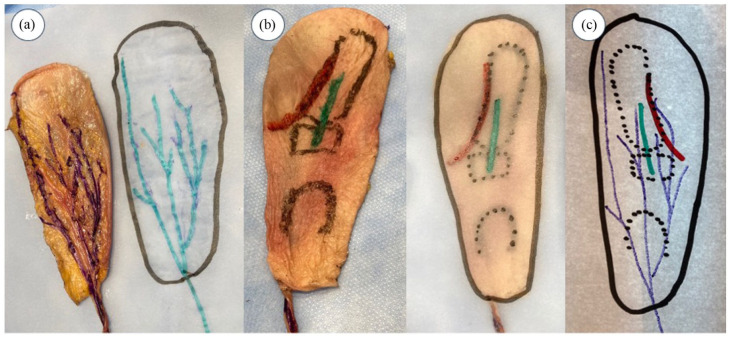
Procedure for studying the topographic relationship between the radial nerve superficial branch (RNSB) and the anterolateral and posterior incisions. *Note.* (a) Tracing of the back of the skin and distribution of the RNSB on transparent paper. (b) Tracing of the skin and approaches on transparent paper. (c) Superposition of the 2 tracings to measure the distance between incisions and RNSB.

Superimposed images of the 1CMCJ, RNSB, and incisions were drawn on paper for each specimen (Figure 2). Two independent examiners (IPGV and NMM) used a millimeter ruler to measure the minimal distance from the incisions to the RNSB.

### Histomorphological Study

A skin sample from each incision (anterolateral and dorsal) was taken from 10 forearms for histologic study. After fixing each sample in 10% buffered formalin for 48 hours at room temperature, it was divided into 2 proximal sections (P1 and P2) and 2 distal sections (D1 and D2) (Figure 1c). These were paraffin-embedded using an automatic tissue processor (model TP1020, Leica Biosystems, Danvers, Massachusetts) and then cut into 4-μm longitudinal sections with a manual microtome (Minot HistoCore-BICOUT, 149BIO000 C1, Leica Biosystems). Tissue sections were deparaffined and rehydrated in successive baths of xylol, 100% ethanol, 95% ethanol, 75% ethanol, 50% ethanol, and distilled water. They were then stained with hematoxylin-eosin and Masson’s trichrome.

**Figure 3.**
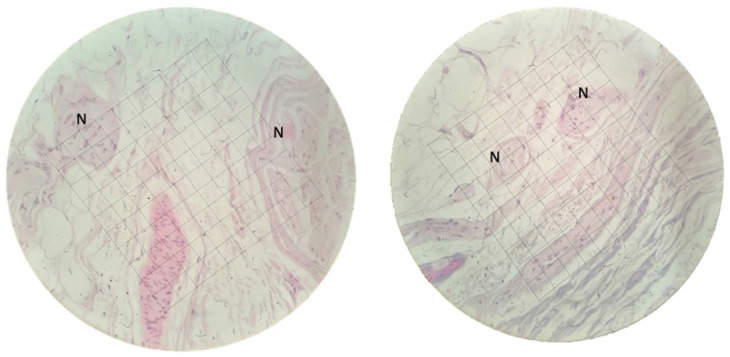
Count of nerve elements on a millimetric scale in the ×40 objective lens of the microscope. *Note.* N = nerve structure.

The total number of nerve endings was counted in each skin sample, calculating the density (per mm^2^). A millimetric scale in the ×40 objective lens of an Olympus BH 2 microscope (Olympus Optical Company, Tokyo, Japan) was used to count the nerve structures in each sample (Figure 3). Counts were performed by 2 independent researchers (IPGV and JTU), using the mean values obtained. Results were expressed as nerve endings/mm^2^ according to the following formula:

$$\begin{array}{l} {\mathrm{number}\;\mathrm{of}\;\mathrm{nerve}\;\mathrm{fascicles/mm}}^{2}\\ =\;\left(\begin{array}{l} \mathrm{number}\;\mathrm{of}\;\mathrm{nerve}\;\mathrm{fascicles/}\\ \mathrm{number}\;\mathrm{of}\;\mathrm{fields}\;\mathrm{studied} \end{array}\right)/0.062.\;\\ \mathrm{where}\;0.062\;\mathrm{is}\;\mathrm{the}\;\mathrm{correction}\;\mathrm{factor}\;\mathrm{for}\;\mathrm{the}\;x40\;\mathrm{objective}. \end{array}$$

### Digital Imaging Analysis

**Figure 4.**
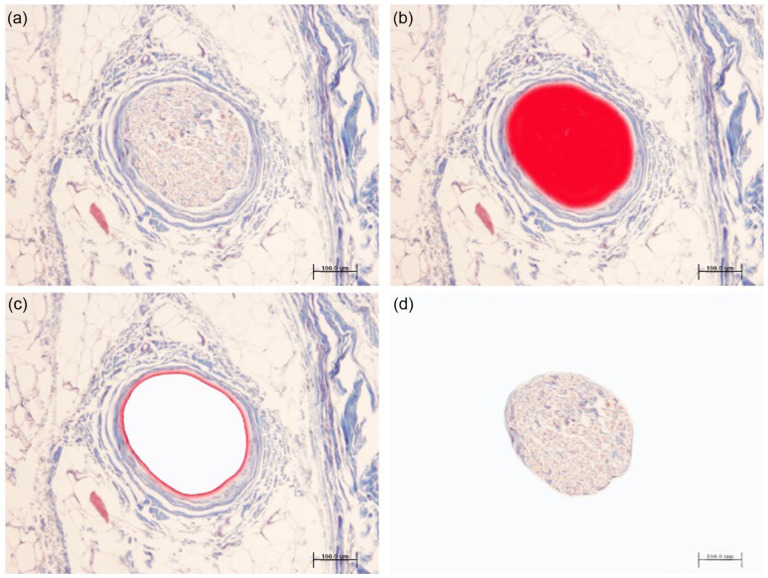
The area occupied by nerve structures was studied under an Olympus BX43 microscope (×20 objective) equipped with an Olympus DP70 digital camera connected to a computer, using ImageJv.1.48. *Note.* The area corresponding to nerve structures was calculated in each sample after capture (a), threshold 24-bit RGB image (b), segmentation (c), and isolated nerve area (d). (Klüver-Barrera stain, original magnification ×20). Scale bar: 100 µm.

The percentage area occupied by nerve structures was studied under an Olympus BX43 microscope (×20 objective) equipped with an Olympus DP70 digital camera connected to a computer, using ImageJv. 1.48 software (http://rsb.info.nih.gov/ij/). The total sample area was measured with a Nikon camera coupled to a Zeiss Axiophot microscope (×1.5 objective), using the ImageJv program. The percentage area corresponding to nerve structures was calculated in each sample (Figure 4).

### Statistical Analysis

SPSS for Windows version 23.0 (IBM SPSS Inc.) was used for statistical analyses. The number and density of nerve endings and the percentage surface area occupied by the nerve were expressed as means with standard deviations. Application of the Kolmogorov-Smirnov test confirmed the normal distribution of quantitative variables. The Student *t* test was used to compare between the 2 sections, and the χ^2^ test was used for qualitative variables. *P* value less than .05 was considered significant in all tests.

## Results

1. *Anatomical study.* Table 1 exhibits the results of the anatomical study. The RNSB was defined as Pattern A if it had 2 branches and Pattern B if it had 3 or more branches. The RNSB branch pattern type had no influence on the frequency of intersections with either anterolateral or dorsal approaches (Student *t* test; *P* = .527). There was no statistically significant difference between approaches in the number of intersections with the RNSB (Student *t* test; *P* = .325). However, the minimum distance to the RNSB was significantly lower (Student *t* test; *P* = .035) with the anterolateral versus dorsal approach (Table 1).
2. *Histomorphometry. Nerve quantification and total density of nerve endings*. Table 2 displays the results obtained. The total number of nerves and the density of nerve endings were significantly higher with the anterolateral versus dorsal approach (Student t test; *P* < .001 for both) (Table 3). The number of nerves, density of nerve endings, and percentage area occupied by nerve structures did not differ between the distal and proximal sections with the dorsal approach (Student t test; *P* = .954, *P* = .892, *P* = .776, respectively) or with the anterolateral approach, although the differences in density and percentage area were close-to-significantly higher in distal versus proximal sections with the latter (Student t test; *P* = .101, *P* = .541, *P* = .068, respectively) (Table 3).

**Table 1.** Results of the Anatomical Study.

Cases	Number of main branches	Number of secondary branches	Branch pattern	Intersects with lateral incision	Intersects with dorsal incision	Mean distance to anterolateral incision (mm)	Mean distance to dorsal incision (mm)
1	3	4	B	No	No	3	6
2	2	5	A	Yes	No	0	6
3	2	4	A	Yes	No	0	5
4	2	4	A	No	No	1	3
5	2	6	A	No	Yes	6	0
6	3	4	B	No	No	2	6
7	2	3	A	No	Yes	1	0
8	2	4	A	Yes	No	0	6
9	2	3	A	Yes	No	0	5
10	2	3	A	Yes	Yes	0	0
Statistical analysis	Minimal distance between surgical incision and radial nerve superficial branches (mm)	Surgical approach	N	Mean	Standard deviation	Typical error	Student *t* test
		Dorsal	10	3.7000	2.71006	0.85700	
		Anterolateral	10	1.3000	1.94651	0.61554	*P* = .035

*Note.* Pattern A: Division in 2 main branches. Pattern B: Division in 3 or more main branches.

**Table 2.** Results of the Histomorphometric and Digital Imaging Analyses.

Approach	Area	Number of nerve structures per skin sample	Density (number of nerves/mm^2^)	Summed nerve area (µm^2^)	Area percentage
Dorsal approach	DP1	10.2	1.3387	175397.979	1.005
	DP2	9.4	1.258	172401.465	0.991
	DD1	9.9	1.3065	153638.76	0.622
	DD2	10.4	1.2903	182389.226	0.861
Anterolateral approach	AP1	13.5	1.8224	105207.813	0.468
	AP2	13.5	1.887	742561.748	2.73
	AD1	19.6	2.29	784856.993	2.616
	AD2	19.8	2.0322	203629.366	1.101

*Note.* DP1 = first proximal segment in dorsal approach; DP2 = second proximal segment in dorsal approach; DD1 = first distal segment in dorsal approach; DD2 = second distal segment in dorsal approach; AP1 = first proximal segment in anterolateral approach; AP2 = second proximal segment in anterolateral approach; AD1 = first distal segment in anterolateral approach; AD2 = second distal segment in anterolateral approach.

**Table 3.** Comparison of the Total Number of Nerves, Nerve Ending Density (Number of Nerve Structures Per mm^2^), and Percentage Area Occupied by Nerve Structures Between Anterolateral and Dorsal Incisions.

Variable	Surgical approach	N	Mean	Standard deviation	Typical error	Student *t* test
Number of nerve structures	Dorsal	40	9.9750	7.51575	1.34536	*P* < .001
	Anterolateral	40	16.6000	8.50882	1.34536	
Density (number of nerve endings/mm^2^)	Dorsal	40	1.2984	0.77995	0.12332	*P* < .001
	Anterolateral	40	2.0080	0.83769	0.13245	
Proximal vs distal segments	Area	N	Mean	Standard deviation	Typical error	Student *t* test
Number of nerve structures	DD2	10	10.4000	8.60491	2.72111	*P* = .954
	DP1	10	10.2000	6.49444	2.05372	
	AD2	10	19.8000	8.79141	2.78009	*P* = .101
	AP1	10	13.5000	7.45729	2.35820	
Density (number of nerve endings/mm^2^)	DD2	10	1.2903	0.89642	0.28347	*P* = .892
	DP1	10	1.3387	0.64991	0.20552	
	AD2	10	2.0322	0.76881	0.24312	*P* = .541
	AP1	10	1.8224	0.73739	0.23318	
Percentage of the area	DD2	10	0.8610	1.16578	0.36865	*P* = .776
	DP1	10	1.0049	1.06253	0.33600	
	AD2	10	1.1014	0.98705	0.31213	*P* = .068
	AP1	10	0.4676	0.30571	0.09667	

*Note.* DD2 = second distal segment in dorsal approach; DP1 = first proximal segment in dorsal approach; AD2 = second distal segment in anterolateral approach; AP1 = first proximal segment in anterolateral approach.

## Discussion

The optimal surgical approach offers the best view and exposure of the structures to be treated while posing the lowest risk of injury to adjacent tissues. To our best knowledge, this study provides the first comparison of this risk between the dorsal and anteroposterior approach to the 1CMCJ, based on anatomical examination of the RNSB and histomorphometric study of the nerve density at incision sites. The main finding was that the minimum distance between incision and RNSB was significantly lower with the anterolateral versus dorsal approach and the number of nerves and density of endings were significantly higher. In addition, the density of nerve endings and the percentage area corresponding to nerve structures appeared to be higher in distal versus proximal sections with the anterolateral approach, although the differences were only close to significant, possibly due to the limited sample size. With the dorsal approach, distal and proximal sections did not differ in number of nerves, nerve ending density, or percentage area occupied by nerve structures, which may suggest that the dorsal incision could be distally extended without increasing the risk of RNSB damage. These findings differ from the observations by Belcher and Nicholl^12^ and Ritchie and Belcher^13^ of a higher frequency of complications with dorsal versus lateral incisions.

Most anatomical studies designed to identify the surgical incision trajectory with the lowest risk of nerve injury have been based on macro- and microscopic dissection of peripheral nerves in the areas of interest.^14-22^ The main strength of the present investigation was the incorporation of histomorphometric and digital imaging analyses to evaluate not only the number and distribution of nerves but also their density and size.

First carpometacarpal joint innervation is known to be based on the RNSB,^23^ and a study of 10 cadavers by Lorea et al^24^ found that it also involves thenar and palmar cutaneous branches of the median nerve and the external cutaneous antebrachial nerve (ECAN). Connections between the RNSB and ECAN were also described by Abrams et al,^25^ Ikiz and Üçerler,^26^ and Mackinnon and Dellon^27^ (reporting a high frequency [70%] of communicating branches). Although Robson et al^28^ highlighted the relationship between the RNSB and the cephalic vein, we do not believe that this negatively impacts on 1CMCJ surgery outcomes.

The present findings are in line with previous reports of the entry of the RNSB into the subcutaneous layer at a mean of 9.0 cm proximal to the radial styloid between the tendons of the brachioradialis and the extensor carpi radialis longus^25^ and of its bifurcation into 2 major branches at approximately 5 cm proximal to the radial styloid.^25,26^,^28-30^

In the present study, 8 out of 10 specimens had 2 main RNSB branches and the other 2 had 3 or more branches. Likewise, a study of 48 arms by Ikiz and Üçerler^26^ found that the RNSB had 2 main branches in 44 arms (91.67%) and 3 main branches in only 4 (8.33%), while Abrams et al^25^ observed 3 main branches in only 15% of cases. At any rate, the risk of intersection did not appear to be influenced by the RNSB distribution pattern in either surgical approach under study.

Widely varied reports have been published on the incidence of neuropathic wound pain after 1CMCJ surgery, and no definitive criteria have been established. Kleinman and Eckenrode^31^ studied 40 patients who underwent trapeziectomy via an anterolateral approach and described persistent wound pain in 2 of these, including one with iatrogenic RNSB injury. In a follow-up study by Dhar et al,^32^ pain was reported in 11 out of 39 hands treated 6 years earlier with a simple trapeziectomy. Belcher and Nicholl^12^ observed neuroma and reduced sensitivity in 4 out of 43 patients at 13 months after trapeziectomy via a dorsal approach. Davis et al^33^ studied 62 patients who also underwent trapeziectomy via a dorsal approach and described wound pain in 5 and symptoms of RNSB dysfunction in 12 at 3 months, although these complications persisted in only 6 patients at 1-year postsurgery. In a retrospective study of 100 thumbs, Weilby^34^ reported 3 permanent and 5 transient cases of RNSB injury after trapeziectomy using a modification of Wagner’s anterior approach with an oblique incision over the thenar eminence. There is a need for prospective studies with strict diagnostic criteria to establish the frequency of RNSB injury in these cases.

Durand et al^35^ explored the relationship between neurovascular structures and incisions in arthroscopic trapeziectomy with tenosuspension. They examined 3 approaches in 15 cadavers with thumb basal joint arthritis: an ulnar portal at the ulnar border of extensor pollicis brevis tendon, a radial portal at the midline between flexor carpi radialis tendon and the abductor pollicis longus, and a novel transosseous portal at the base of the first metacarpal (“trans-M1” approach). Neurovascular structures at risk were found to be the radial artery with the ulnar portal, branches of the superficial branch of the radial nerve with all portals, and the ending of the lateral cutaneous nerve of the forearm with the radial and “trans-M1” approaches. Other authors have reported a high risk of nerve injury with approaches at the radial border of the abductor pollicis longus^36,37^ or radial margin of the carpal radial flexor tendon.^38^ The present results are in line with these findings, observing a larger number of nerve endings with the anterolateral approach, corresponding to the most radial area of the 1CMCJ, than with the dorsal approach.

Knowledge of the course of the RNSB helps to prevent injury in 1CMCJ surgery. The dorsal approach appears to be safer than the anterolateral approach because the incision is located between the 2 branches of the “V” formed by the division of the RNSB, and it can be extended distally if necessary, without increasing the risk of nerve injury. However, the dorsal approach does not guarantee avoiding injury to the RNSB, and careful dissection is still necessary even if the incision location seems more favorable.

The main study limitations are the small sample size and the utilization of cryopreserved specimens, given that *postmortem* loss of tissue tone can alter anatomical relationships and there is no clinical outcome. In addition, analog methods were used for macroscopic measurements, and the digital imaging analyses were semi-automatized, although the examiner selected the area for evaluation.

## Conclusions

The incision is closer to the RNSB pathway with Wagner’s anterolateral approach than with Gervis’ dorsal approach, crossing with nerve branches in 50% of cases. The density of nerves is higher in the skin overlying the anterolateral versus dorsal aspect of the carpometacarpal joint. The risk of neuropathic wound pain after carpometacarpal joint surgery could be higher with the anterolateral approach, which should therefore be avoided.

## Supplemental Material

sj-docx-1-han-10.1177_15589447251392940 – Supplemental material for Risk of Injury to the Superficial Branch of the Radial Nerve in Dorsal and Anterolateral Approaches to the First Carpometacarpal Joint Supplemental material, sj-docx-1-han-10.1177_15589447251392940 for Risk of Injury to the Superficial Branch of the Radial Nerve in Dorsal and Anterolateral Approaches to the First Carpometacarpal Joint by Irene Pérez de Gracia-Velázquez, Javier De Torres-Urrea, Olga Roda, Clarisa Simón-Pérez, Natividad Martín-Morales, Francisco O’Valle and Pedro Hernández-Cortés in HAND

## References

[1] Shuler MS Luria S Trumble TE. Basal joint arthritis of the thumb. J Am Acad Orthop Surg. 2008;16(7):418-423. 18611999 10.5435/00124635-200807000-00007

[2] Zollinger PE Unal H Ellis ML , et al. Clinical results of 40 consecutive basal thumb prostheses and no CRPS type I after vitamin C prophylaxis. Open Orthop J. 2010;4:62-66. 20224742 10.2174/1874325001004020062 PMC2835870

[3] Ropars M Fontaine I Morandi X , et al. Preserving the superficial branch of the radial nerve during carpometacarpal and metacarpophalangeal joint arthroscopy: an anatomical study. Surg Radiol Anat. 2010;32(3):271-276. 20082078 10.1007/s00276-010-0622-8

[4] Bricout M Rezzouk J. Complications and failures of the trapeziometacarpal Maia® prosthesis: a series of 156 cases. Hand Surg Rehabil. 2016;35(3):190-198. 27740461 10.1016/j.hansur.2016.02.005

[5] Saab M Chick G. Trapeziectomy for trapeziometacarpal osteoarthritis. Bone Jt Open. 2021;2(3):141-149. 33650434 10.1302/2633-1462.23.BJO-2020-0188.R1 PMC8009903

[6] Tchurukdichian A Lussiez B. Surgical approaches to the trapeziometacarpal joint for the insertion of implants and prostheses (non-arthroscopy). Hand Surg Rehabil. 2021;40S:S29-S32. 10.1016/j.hansur.2020.10.019 33581362

[7] Gervis WH. Excision of the trapezium for osteoarthritis of the trapezio-metacarpal joint. J Bone Joint Surg Br. 1949;31B(4):537-539. 15397137

[8] Teissier J. La voie d’abord latérale: voie royale de la prothèse trapézo-métacarpienne. Chir Main. 2011;30(suppl 1):95-97.

[9] Wagner CJ. Method of treatment of Bennett’s fracture dislocation. Am J Surg. 1950;80(2):230-231. 15425714 10.1016/0002-9610(50)90537-x

[10] McHanwell S Brenner E Chirculescu ARM , et al. The legal and ethical framework governing body donation in Europe—a review of current practice and recommendations for good practice. Eur J Anat. 2008;12(1):1-24.

[11] Riederer BM. Body donations today and tomorrow: what is best practice and why? Clin Anat. 2016;29(1):11-18. 26475613 10.1002/ca.22641

[12] Belcher HJ Nicholl JE. A comparison of trapeziectomy with and without ligament reconstruction and tendon interposition. J Hand Surg Br. 2000;25(4):350-356. 11058002 10.1054/jhsb.2000.0431

[13] Ritchie JF Belcher HJ. A comparison of trapeziectomy via anterior and posterior approaches. J Hand Surg Eur Vol. 2008;33(2):137-143. 18443051 10.1177/1753193407087571

[14] Bezerra AJ Carvalho VC Nucci A. An anatomical study of the palmar cutaneous branch of the median nerve. Surg Radiol Anat. 1986;8(3):183-188. 3099409 10.1007/BF02427847

[15] Born T Mahoney J. Cutaneous distribution of the ulnar nerve in the palm: does it cross the incision used in carpal tunnel release? Ann Plast Surg. 1995;35(1):23-25. 7574281 10.1097/00000637-199507000-00005

[16] Hobbs RA Magnussen PA Tonkin MA. Palmar cutaneous branch of the median nerve. J Hand Surg Am. 1990;15(1):38-43. 2299166 10.1016/s0363-5023(09)91103-0

[17] Martin CH Seiler JG 3rd Lesesne JS. The cutaneous innervation of the palm: an anatomic study of the ulnar and median nerves. J Hand Surg Am. 1996;21(4):634-638. 8842957 10.1016/S0363-5023(96)80017-7

[18] Matloub HS Yan JG Mink Van Der Molen AB , et al. The detailed anatomy of the palmar cutaneous nerves and its clinical implications. J Hand Surg Br. 1998;23(3):373-379. 9665529 10.1016/s0266-7681(98)80061-2

[19] Ozcanli H Coskun NK Cengiz M , et al. Definition of a safe-zone in open carpal tunnel surgery: a cadaver study. Surg Radiol Anat. 2010;32(3):203-206. 19337677 10.1007/s00276-009-0498-7

[20] Tubbs RS Rogers JM Loukas M , et al. Anatomy of the palmar branch of the ulnar nerve: application to ulnar and median nerve decompressive surgery. J Neurosurg. 2011;114(1):263-267. 20367079 10.3171/2010.3.JNS091249

[21] Natsis K Karanassos MT Papathanasiou E , et al. Coexisting anatomical variation of median and ulnar nerves in a cadaver palm. Folia Morphol (Warsz). 2012;71(4):269-274. 23197148

[22] Sulaiman S Soames R Lamb C. Ulnar nerve cutaneous distribution in the palm: application to surgery of the hand. Clin Anat. 2015;28(8):1022-1028. 26378873 10.1002/ca.22626

[23] Cozzi EP . Dénervation des articulations du poignet et de la main. In: Tubiana R , ed. Traité de chirurgie de la main (Vol. 4). Masson; 1991:781-787.

[24] Lorea DP Berthe JV De Mey A , et al. The nerve supply of the trapeziometacarpal joint. J Hand Surg Br. 2002;27(3):232-237. 12074608 10.1054/jhsb.2001.0716

[25] Abrams RA Brown RA Botte MJ. The superficial branch of the radial nerve: an anatomic study with surgical implications. J Hand Surg Am. 1992;17(6):1037-1041. 1430933 10.1016/s0363-5023(09)91056-5

[26] Ikiz ZA Uçerler H. Anatomic characteristics and clinical importance of the superficial branch of the radial nerve. Surg Radiol Anat. 2004;26(6):453-458. 15365770 10.1007/s00276-004-0256-9

[27] Mackinnon SE Dellon AL. The overlap pattern of the lateral antebrachial cutaneous nerve and the superficial branch of the radial nerve. J Hand Surg Am. 1985;10(4):522-526. 4020063 10.1016/s0363-5023(85)80076-9

[28] Robson AJ See MS Ellis H. Applied anatomy of the superficial branch of the radial nerve. Clin Anat. 2008;21(1):38-45. 18092362 10.1002/ca.20576

[29] Samarakoon LB Lakmal KC Thillainathan S , et al. Anatomical relations of the superficial sensory branches of the radial nerve: a cadaveric study with clinical implications. Patient Saf Surg. 2011;5(1):28. 22054296 10.1186/1754-9493-5-28 PMC3269985

[30] Bridgwater H Mertz T Brassett C , et al. Systematic review of nerves at risk at the wrist in common surgical approaches to the forearm: anatomical variations and surgical implications. Clin Anat. 2024;37(4):425-439. 38059329 10.1002/ca.24129

[31] Kleinman WB Eckenrode JF. Tendon suspension sling arthroplasty trapeziometacarpal arthritis. J Hand Surg Am. 1991;16(6):983-991. 1748769 10.1016/s0363-5023(10)80056-5

[32] Dhar S Gray IC Jones WA , et al. Simple excision of the trapezium for osteoarthritis of the carpometacarpal joint of the thumb. J Hand Surg Br. 1994;19(4):485-488. 7964101 10.1016/0266-7681(94)90214-3

[33] Davis TR Brady O Dias JJ. Excision of the trapezium for osteoarthritis of the trapeziometacarpal joint: a study of the benefit of ligament reconstruction or tendon interposition. J Hand Surg Am. 2004;29(6):1069-1077. 15576217 10.1016/j.jhsa.2004.06.017

[34] Weilby A. Tendon interposition arthroplasty of the first carpo-metacarpal joint. J Hand Surg Br. 1988;13(4):421-425. 3249143 10.1016/0266-7681_88_90171-4

[35] Durand S Gagey O Masquelet AC , et al. Neurovascular relationships of the approaches for arthroscopic total trapeziectomy with ligamentous stabilization. Surg Radiol Anat. 2005;27(3):165-170. 15744448 10.1007/s00276-004-0310-7

[36] Berger RA. A technique for arthroscopic evaluation of the first carpometacarpal joint. J Hand Surg Am. 1997;22(6):1077-1080. 9471080 10.1016/S0363-5023(97)80052-4

[37] Gonzalez MH Kemmler J Weinzweig N , et al. Portals for arthroscopy of the trapeziometacarpal joint. J Hand Surg Br. 1997;22(5):574-575. 9752905 10.1016/s0266-7681(97)80347-6

[38] Orellana MA Chow JC. Arthroscopic visualization of the thumb carpometacarpal joint: introduction and evaluation of a new radial portal. Arthroscopy. 2003;19(6):583-591. 12861196 10.1016/s0749-8063(03)00119-1

