# Supplementary material for: Risk of Injury to the Superficial Branch of the Radial Nerve in Dorsal and Anterolateral Approaches to the First Carpometacarpal Joint
Source: Hand (N Y). 2025 Dec 17:15589447251392940. Online ahead of print. doi: 10.1177/15589447251392940 (PMC12711530; doi:10.1177/15589447251392940)
Supplement: sj-docx-1-han-10.1177_15589447251392940 – Supplemental material for Risk of Injury to the Superficial Branch of the Radial Nerve in Dorsal and Anterolateral Approaches to the First Carpometacarpal Joint [file sj-docx-1-han-10.1177_15589447251392940.docx]

Suplementary material 1

| Cases | Number of nerves | Density (nº nerves /mm^2^) | Total area (µm^2^) | Summed nerve area (µm^2^) | % |
| --- | --- | --- | --- | --- | --- |
| 1 anterior distal 1 | 22 | 2.097 | 20565898.389 | 43196.582 | 0.210 |
| 1 anterior distal 2 | 22 | 1.29 | 27173283.767 | 30039.455 | 0.111 |
| 1 anterior proximal 1 | 12 | 2.742 | 37495662.949 | 39437.110 | 0.105 |
| 1 anterior proximal 2 | 10 | 2.742 | 22672565.056 | 11509.960 | 0.051 |
| 1 dorsal distal 1 | 6 | 0.806 | 26504832.714 | 8165.722 | 0.031 |
| 1 dorsal distal 2 | 11 | 1.129 | 23213779.430 | 6780.470 | 0.029 |
| 1 dorsal proximal 1 | 8 | 1.29 | 24197620.818 | 44893.556 | 0.186 |
| 1 dorsal proximal 2 | 6 | 0.968 | 25431524.164 | 41697.754 | 0.164 |
| 2 anterior distal 1 | 31 | 2.903 | 39297496.902 | 241570.606 | 0.615 |
| 2 anterior distal 2 | 25 | 2.258 | 32029987.608 | 125813.477 | 0.393 |
| 2 anterior proximal 1 | 9 | 1.29 | 30148351.921 | 94484.961 | 0.313 |
| 2 anterior proximal 2 | 16 | 1.935 | 36075588.600 | 61792.187 | 0.171 |
| 2 dorsal distal 1 | 8 | 1.129 | 30183197.026 | 275328.321 | 0.912 |
| 2 dorsal distal 2 | 5 | 0.806 | 23661214.374 | 222417.774 | 0.940 |
| 2 dorsal proximal 1 | 7 | 1.129 | 26758711.276 | 87733.593 | 0.328 |
| 2 dorsal proximal 2 | 3 | 0.484 | 29623990.087 | 9757.813 | 0.033 |
| 3 anterior distal 1 | 13 | 1.613 | 7633209.418 | 43753.711 | 0.573 |
| 3 anterior distal 2 | 9 | 1.29 | 4904997.935 | 35641.896 | 0.727 |
| 3 anterior proximal 1 | 15 | 1.935 | 11641239.157 | 66612.404 | 0.572 |
| 3 anterior proximal 2 | 24 | 2.419 | 13207335.812 | 174747.557 | 1.323 |
| 3 dorsal distal 1 | 10 | 1.452 | 10805204.461 | 141468.847 | 1.309 |
| 3 dorsal distal 2 | 13 | 2.258 | 14282379.182 | 134440.138 | 0.941 |
| 3 dorsal proximal 1 | 8 | 1.29 | 7847236.679 | 236108.302 | 3.009 |
| 3 dorsal proximal 2 | 6 | 0.645 | 5693895.085 | 181927.540 | 3.195 |
| 4 anterior distal 1 | 11 | 1.774 | 16602973.978 | 199053.811 | 1.199 |
| 4 anterior distal 2 | 6 | 0.968 | 6702354.399 | 48719.335 | 0.727 |
| 4 anterior proximal 1 | 13 | 1.935 | 47413581.165 | 99003.027 | 0.209 |
| 4 anterior proximal 2 | 7 | 0.968 | 15303543.990 | 9900.780 | 0.065 |
| 4 dorsal distal 1 | 4 | 0.645 | 4807980.173 | 16719.629 | 0.348 |
| 4 dorsal distal 2 | 2 | 0.323 | 5043915.737 | 3678.809 | 0.073 |
| 4 dorsal proximal 1 | 12 | 1.452 | 6346666.667 | 21649.415 | 0.341 |
| 4 dorsal proximal 2 | 16 | 2.258 | 9429541.512 | 28107.033 | 0.298 |
| 5 anterior distal 1 | 9 | 1.452 | 24802379.182 | 27497.951 | 0.111 |
| 5 anterior distal 2 | 28 | 2.903 | 22806443.618 | 463848.340 | 2.034 |
| 5 anterior proximal 1 | 14 | 2.258 | 25801140.025 | 121791.797 | 0.472 |
| 5 anterior proximal 2 | 19 | 2.903 | 17740223.048 | 159159.862 | 0.897 |
| 5 dorsal distal 1 | 8 | 1.452 | 22806889.715 | 250234.180 | 1.097 |
| 5 dorsal distal 2 | 3 | 0.484 | 13829194.548 | 5575.849 | 0.040 |
| 5 dorsal proximal 1 | 3 | 0.484 | 8228946.716 | 18028.027 | 0.219 |
| 5 dorsal proximal 2 | 6 | 0.968 | 14686195.787 | 28158.984 | 0.192 |
| 6 anterior distal 1 | 16 | 2.097 | 33352465.923 | 6762695.216 | 20.276 |
| 6 anterior distal 2 | 14 | 1.774 | 20501858.736 | 644403.517 | 3.143 |
| 6 anterior proximal 1 | 4 | 0.645 | 34103742.256 | 267787.207 | 0.785 |
| 6 anterior proximal 2 | 20 | 2.097 | 29663048.327 | 6690056.838 | 22.554 |
| 6 dorsal distal 1 | 6 | 1.129 | 24354250.310 | 117546.095 | 0.483 |
| 6 dorsal distal 2 | 7 | 1.129 | 27049764.560 | 118477.442 | 0.438 |
| 6 dorsal proximal 1 | 1 | 0.161 | 37414721.190 | 236953.711 | 0.633 |
| 6 dorsal proximal 2 | 9 | 1.290 | 32272565.056 | 722840.625 | 2.240 |
| 7 anterior distal 1 | 35 | 5.000 | 25229789.343 | 214692.966 | 0.851 |
| 7 anterior distal 2 | 32 | 3.065 | 21426666.667 | 211741.699 | 0.988 |
| 7 anterior proximal 1 | 19 | 2.419 | 27688376.704 | 90178.907 | 0.326 |
| 7 anterior proximal 2 | 7 | 1.129 | 5752465.923 | 26627.540 | 0.463 |
| 7 dorsal distal 1 | 32 | 3.226 | 38169070.632 | 659459.768 | 1.728 |
| 7 dorsal distal 2 | 28 | 2.742 | 28061462.206 | 738135.257 | 2.630 |
| 7 dorsal proximal 1 | 19 | 2.258 | 20834646.840 | 511147.656 | 2.453 |
| 7 dorsal proximal 2 | 25 | 2.581 | 19879785.213 | 226573.339 | 1.140 |
| 8 anterior distal 1 | 24 | 2.258 | 15192019.827 | 91453.518 | 0.602 |
| 8 anterior distal 2 | 16 | 1.774 | 17007038.414 | 91609.374 | 0.539 |
| 8 anterior proximal 1 | 7 | 1.129 | 9592912.020 | 50626.563 | 0.528 |
| 8 anterior proximal 2 | 15 | 1.774 | 16480346.964 | 126264.162 | 0.766 |
| 8 dorsal distal 1 | 13 | 1.613 | 31409863.693 | 31174.806 | 0.099 |
| 8 dorsal distal 2 | 19 | 1.613 | 20254820.322 | 49405.471 | 0.244 |
| 8 dorsal proximal 1 | 22 | 2.097 | 35510582.404 | 277120.120 | 0.780 |
| 8 dorsal proximal 2 | 3 | 0.484 | 10496257.745 | 47202.344 | 0.450 |
| 9 anterior distal 1 | 7 | 1.129 | 8224089.219 | 31466.602 | 0.383 |
| 9 anterior distal 2 | 17 | 1.935 | 21711821.561 | 59388.770 | 0.274 |
| 9 anterior proximal 1 | 11 | 1.129 | 22482230.483 | 54144.434 | 0.241 |
| 9 anterior proximal 2 | 4 | 0.645 | 10471177.200 | 32018.555 | 0.306 |
| 9 dorsal distal 1 | 3 | 0.323 | 12672664.188 | 5960.742 | 0.047 |
| 9 dorsal distal 2 | 1 | 0.161 | 10657001.239 | 456.738 | 0.004 |
| 9 dorsal proximal 1 | 10 | 1.452 | 38725947.955 | 53213.086 | 0.137 |
| 9 dorsal proximal 2 | 2 | 0.323 | 30784337.051 | 4758.789 | 0.015 |
| 10 anterior distal 1 | 28 | 2.581 | 14370210.657 | 193188.964 | 1.344 |
| 10 anterior distal 2 | 29 | 3.065 | 15646641.884 | 325087.796 | 2.078 |
| 10 anterior proximal 1 | 31 | 2.742 | 14933333.333 | 168011.721 | 1.125 |
| 10 anterior proximal 2 | 13 | 2.258 | 18824287.485 | 133540.039 | 0.709 |
| 10 dorsal distal 1 | 9 | 1.29 | 18738339.529 | 30329.492 | 0.162 |
| 10 dorsal distal 2 | 15 | 2.258 | 16646592.317 | 544524.316 | 3.271 |
| 10 dorsal proximal 1 | 12 | 1.774 | 13607087.980 | 267132.324 | 1.963 |
| 10 dorsal proximal 2 | 18 | 2.581 | 19834448.575 | 432990.430 | 2.183 |
